# Polyphenols and health: Interactions between fibre, plant polyphenols and the gut microbiota

**DOI:** 10.1111/nbu.12296

**Published:** 2017-11-10

**Authors:** C. A. Edwards, J. Havlik, W. Cong, W. Mullen, T. Preston, D. J. Morrison, E. Combet

**Affiliations:** ^1^ University of Glasgow Glasgow UK

**Keywords:** bioavailability, dietary fibre, gut microbiota, phenolic acids, polyphenols

## Abstract

A high‐fibre diet and one rich in fruit and vegetables have long been associated with lower risk of chronic disease. There are several possible mechanisms underpinning these associations, but one likely important factor is the production of bioactive molecules from plant‐based foods by the bacteria in the colon. This links to our growing understanding of the role of the gut microbiome in promoting health. Polyphenolic‐rich plant foods have been associated with potential health effects in many studies, but the bioavailability of polyphenol compounds, as eaten, is often very low. Most of the ingested molecules enter the large intestine where they are catabolised to smaller phenolic acids that may be the key bioactive effectors. Dietary fibres, present in plant foods, are also fermented by the bacteria to short‐chain fatty acids, compounds associated with several beneficial effects on cell turnover, metabolism and eating behaviour. Polyphenols and fibre are often eaten together, but there is a lack of research investigating the interaction between these two groups of key substrates for the colonic bacteria. In a project funded by the Biotechnology and Biological Sciences Research Council Diet and Health Research Industry Club, we are investigating whether combining different fibres and polyphenol sources can enhance the production of bioactive phenolic acids to promote health. This could lead to improved dietary recommendations and to new products with enhanced potential health‐promoting actions.

## Introduction

There has been intense interest in the role of the gut microbiome in human health over the last decade. The ability to describe the genetic repertoire of bacterial populations without the need to isolate, culture and characterise each organism has revolutionised our ability to understand the complexity of this important ecosystem. It is becoming increasingly evident that our gut bacteria have important influences on several functions of the human body. The diversity and composition of the gut microbiota has been associated with a wide variety of disorders and pathologies including obesity (Turnbaugh *et al*. 2006; Khan *et al*. 2016), inflammatory bowel disease (Quince *et al*. 2015), autoimmune diseases (Maeda & Takeda 2017), allergy (Lynch & Boushey 2016) and neurological disorders (Wang & Kasper 2014). Our understanding of the mechanisms underpinning interactions between the gut microbiota and human systems is, however, still limited. It is becoming clear that many host‐microbe effects are mediated through the release of bioactive molecules by bacteria in the gut and absorption of these metabolites into the circulation. Two key groups of such metabolites are phenolic acids (from a wide variety of plant polyphenols) and short‐chain fatty acids (SCFA), from the fermentation of non‐digestible carbohydrates, a major component of dietary fibre. Fibre itself can be an important source of non‐extractible polyphenols (Saura‐Calixto *et al*. 2010). While we understand well how dietary polyphenols and non‐digestible carbohydrates are metabolised by the gut microbiota at substrate level, curiously, very little attention has been paid to how these two key groups of microbial substrate interact, given that they are often consumed together and that the catabolism of one may affect the catabolism of the other.

## Gut bacteria and polyphenols

Polyphenols are complex compounds in fruit and vegetables that help protect the plant from damage, for example from UV radiation and pathogens; they often confer the vibrant colours associated with fruit and vegetables. Polyphenols have chemical structures based on hydroxylated phenolic rings. They are usually eaten in plant‐based foods in a glycosylated form – with a sugar attached to the main polyphenolic structure. Polyphenols are classified into a range of structurally related groups, with over 9000 different structures described in the flavonoid group alone. Average intake is approximately 1 g/day (Pérez‐Jiménez *et al*. 2011). Zamora‐Ros *et al*. (2016) reported details of dietary polyphenol intakes from the *European Prospective Investigation into Cancer and Nutrition* (*EPIC*) study. In Mediterranean countries, flavonoids were the main polyphenolic contributors but were only the second contributor in non‐Mediterranean Europe where phenolic acids were the main contributor. In Mediterranean countries, the main food sources were coffee, fruit and then wine. In non‐Mediterranean countries, the order of contribution was coffee, tea and then fruit. Fruit was the main source of flavonoids in Mediterranean countries, whereas tea was the main source in the non‐Mediterranean countries. Flavonoids include flavonols, flavones, flavanones, isoflavones, flavanols and anthocyanins. However, most of these parent polyphenols are not well absorbed in the small intestine (Williamson & Manach 2005) and over 90% enter the large intestine where they are catabolised by the colonic microbiota (Ozdal *et al*. 2016). Thus, the bioavailability of the parent compounds is very low. The microbiota degrades the parent polyphenolics to a range of intermediates and end products including phenolic acids, such as 3‐hydroxyphenylacetic acid, from the metabolism of the flavonol rutin (found in tomatoes, for example). Evidence is emerging regarding the health benefits of these intermediates and end products, with the realisation that their high bioavailability (relative to that of the parent compounds) may explain many of the biological effects previously attributed to the polyphenolics (Russell & Duthie 2011). Phenolic acids can be detected in both plasma and urine after a meal, but some also result from mammalian processes (*e.g*. protein catabolism) thus complicating the interpretation of plasma and urine phenolic acid measurements. One of the main intermediate metabolites of rutin catabolism, 3,4–dihydroxy phenyl acetic acid (3,4DHPAA), exhibited greater inhibition of anti‐platelet aggregation (Kim *et al*. 1998) and secretion of proinflammatory cytokines TNF‐α and IL‐6 in monocytes (Monagas *et al*. 2009) than the parent compound. Phenolic acids have also been shown to inhibit protein glycation (Pashikanti *et al*. 2010; Vlassopoulos *et al*. 2015). Most studies assessing phenolic acid bioactivity, however, are still based on *in vitro* models and more *in vivo* evidence is needed.

## Bioactive molecules from dietary fibre

Another major component of many plant‐based foods is dietary fibre. The current definition of dietary fibre includes carbohydrate polymers and oligosaccharides that are not hydrolysed by the endogenous enzymes in the small intestine of humans (Jones 2013). These carbohydrates are available for fermentation by the colonic microbiota. The main products of colonic fermentation are SCFA: mainly acetic, propionic and butyric acids and gases (carbon dioxide, hydrogen, methane and, in some individuals, hydrogen sulphide). Some SCFA can be produced by bacteria from protein degradation, while acetate is also produced by mammalian metabolism. This complicates the interpretation of plasma SCFA and makes measurement of colonic production of SCFA from non‐digestible carbohydrates *in vivo* problematic. Stable isotope methods, feeding isotopically labelled compounds (Verbeke *et al*. 2010) or measuring the dilution of isotopically labelled SCFA infused into the blood (Boets *et al*. 2017), have begun to make *in vivo* colonic SCFA production measurements possible.

SCFA have been associated with a range of potential health benefits and act as the natural ligands for free fatty acid receptors (FFAR 2/3), which are expressed on a wide array of cell types (Nøhr *et al*. 2013). This has led to renewed interest in the link between dietary fibre and health, mediated by SCFA (reviewed in Morrison & Preston 2016). Of the SCFA, butyrate has been shown to be the preferred fuel of colonocytes (Roediger 1982) but also stimulates apoptosis and cell differentiation of cancer cells *in vitro* (Hague & Paraskeva 1995) and promotes mucosal healing from inflammation (Vernia *et al*. 2003). Propionate has been shown to stimulate the release of satiety hormones, such as PYY and GLP‐1, influencing appetite regulation (Chambers *et al*. 2015) and glucose metabolism (Pingitore *et al*. 2017).

## Interactions between polyphenols and dietary fibre

Fibre could influence the bacterial catabolism of polyphenols by several mechanisms, depending on the nature of the fibre. Some fibres may entrap polyphenols in the lumen of the gut; this could be physical sequestration in a viscous environment or physicochemical binding in a plant cell matrix reducing their absorption in the small intestine and increasing their bioavailability for bacterial catabolism (Perez‐Jimenez *et al*. 2013; Renard *et al*. 2017). Fibres themselves are fermented and may selectively increase the activity of bacteria that positively or negatively influence those responsible for polyphenol catabolism (Tzounis *et al*. 2008). The supply of fermentable fibre may alter bacterial activity away from polyphenol catabolism, a phenomenon observed with protein catabolism; fibre fermentation appears to lead to a reduction in protein–amino acid catabolism (François *et al*. 2012), probably resulting in greater incorporation of dietary protein derived amino acids into bacterial biomass (Windey *et al*. 2015). The production of SCFA from fermentable carbohydrates may reduce the colonic luminal pH to below 5 (Florent *et al*. 1985) depending on the speed of fermentation and the buffering capacity in the colon. This pH may in turn inhibit bacteria responsible for some metabolic activities. The impact of fibre on gut motility and transit time could also influence the site of phenolic acid production and absorption, changing the rate of absorption. This has been shown for different types of fibre and SCFA production (Govers *et al*. 1999; Morita *et al*. 1999) but not yet for polyphenols. Much of the evidence for colonic metabolism of polyphenols has been produced using *in vitro* batch cultures with human stool samples. However, the medium used in these models was usually free from fermentable carbohydrate, leaving the polyphenols as the sole source of carbon (an unusual scenario *in vivo*). In previous pilot studies, we have shown that combining fermentable carbohydrates and polyphenols in an *in vitro* model of colonic fermentation speeded up the breakdown of the polyphenol rutin (Jaganath *et al*. 2006), but had no effect on phenolic acid production from hesperidin (Hou *et al*. 2015). Moreover, a range of fermentable fibres inhibited phenolic acid production from rutin (Mansoorian *et al*. 2015).

In turn, polyphenols could influence the fermentation of the fibre carbohydrates as several polyphenols have been shown to have both anti‐bacterial (Taguri *et al*. 2004) and prebiotic actions (Tuohy *et al*. 2012).

In our project *Manipulating the activity of the gut microbiota with fermentable carbohydrates to maximise the bioavailability of bioactive phenolic acids for health* funded as part of the Biotechnology and Biological Sciences Research Council (BBSRC) Diet and Health Research Industry Club (DRINC) initiative, we are exploring the interactions between dietary fibres and colonic polyphenol catabolism in a systematic fashion. Starting with *in vitro* fermentation models, using human faecal bacteria, a range of fibres has been combined at different doses with a range of polyphenols, so that the relative effects of (1) fibre on individual polyphenol catabolism and (2) the polyphenols on SCFA production can be estimated. The results of these fermentations will then inform the choice of two fibre‐polyphenol mixtures to be studied in acute bioavailability studies in humans. Phenolic acid production will be measured in urine over 24 hours after a test meal. Finally, a 6‐week feeding study will explore the longer term interactive effects of the fibre and polyphenol mixture on phenolic acids and also biomarkers of inflammation and health. We are using stable isotope‐labelled polyphenols and foods to confirm the source of phenolic acids in these studies. Thus, the results of this project should inform clearer dietary recommendations and may lead to new product designs for enhancing the positive actions of the dietary polyphenols.

## Conflict of interest

We have no conflict of interest to declare.
